# Clinical and Histopathological Characteristics in Women With Postmenopausal Bleeding: A Study of 120 Women in a Tertiary Care Hospital in Punjab

**DOI:** 10.7759/cureus.51690

**Published:** 2024-01-05

**Authors:** Shailja Talwar, Harpreet Kaur, Isha Tapasvi, Sarita Nibhoria, Chaitanya Tapasvi

**Affiliations:** 1 Department of Obstetrics and Gynecology, Community Health Center, Khamanon, IND; 2 Department of Obstetrics and Gynecology, Guru Gobind Singh Medical College and Hospital, Faridkot, IND; 3 Department of Pathology, Guru Gobind Singh Medical College and Hospital, Faridkot, IND; 4 Department of Radiodiagnosis, Guru Gobind Singh Medical College and Hospital, Faridkot, IND

**Keywords:** endometrial hyperplasia, menopause, endometrial thickness (et), endometrial cancer (ec), postmenopausal bleeding

## Abstract

Background

Postmenopausal bleeding (PMB) is defined as blood loss from the genital tract occurring 12 months or more after an individual’s last menstrual period. It is important for women to recognize abnormal symptoms during menopause, with PMB being one of the most critical. PMB is a common clinical presentation and can be indicative of endometrial carcinoma. A thorough clinical assessment and endometrial histopathology can ensure early diagnosis and treatment of malignancy in high-risk patients.

Materials and Methods

This study included 120 women with PMB. Their clinical and histopathological characteristics were studied, and correlations between the characteristics were investigated. Patients were evaluated according to their age, parity, duration of menopause, and socioeconomic status. Various comorbidities such as diabetes mellitus, hypertension, and obesity were noted.

Results

The patients ranged in age from 45 to 80 years, with a mean age of 54.97 ± 5.86 years. Fifty-nine (49.16%) of the patients presented with PMB within 3 years of menopause. PMB was seen most commonly in patients with parity 3, accounting for 37 (30.83%) of the cases. Endometrial thickness was increased in 100 (83.33%) cases. The most common causes of PMB were simple hyperplasia without atypia (SHWOA) in 36 (36%) patients and atrophic endometrium in 14 (14%) patients. Twelve (10%) of the patients had endometrial carcinoma. Benign causes of PMB were present in 91 (75.3%) cases, whereas 29 (24.1%) had a malignant cause. Weakly positive, but significant correlations (*P* < 0.05) were seen between the development of malignancy and increasing age (Pearson correlation coefficient, r = 0.263) parity (r = 0.244), and body mass index (r = 0.272).

Conclusions

PMB is considered abnormal. Benign causes are more common, but malignant causes are possible. In the current study, endometrial carcinoma was the most common malignant cause of PMB. Endometrial carcinoma incidence increased with greater endometrial thickness and more years since menopause. Histopathological examination remains the criterion standard for the correct diagnosis. Initiatives are recommended for increasing awareness about PMB to support prompt medical attention for a better prognosis.

## Introduction

Menopause occurs when estrogen levels fall and ovulation ceases, and it is defined by the permanent cessation of menstruation after 1 year from the last menstrual period [1]. It is important for women to recognize the various problems associated with menopause, with postmenopausal bleeding (PMB) being one of the most critical. PMB is defined as blood loss from the genital tract occurring 12 or more months after the last menstrual period [2]. The worldwide prevalence of PMB is approximately 10% [3]. Among the women presenting with PMB, 80%-90% have benign conditions such as endometrial or vaginal atrophy, cervical polyps, endometrial polyps, and decubitus ulcer in case of uterovaginal prolapse, neglected pessary, or a forgotten intrauterine device [4]. Some uncommon benign causes of PMB are chronic endometritis of tuberculosis, thrombocytopenia, leukemia, use of anticoagulants, and secondary coagulopathy from liver disease [5]. Although the most frequent causes of PMB are benign conditions, it is important to exclude malignant causes.

Clinical examination in any PMB case may include a cervical smear test, pelvic sonography to measure the endometrial thickness (ET), diagnostic hysteroscopy, and biopsy from the possible site of bleeding [6]. The American College of Obstetricians and Gynecologists recommends transvaginal ultrasound after a thorough clinical history and examination are completed [1].

Nearly 13% of cases with PMB are due to endometrial carcinoma [7]. Endometrial hyperplasia is defined as a condition with excessive proliferation of the endometrial cells, and endometrial cancer is more likely with increasing ET [1,8]. Risk factors for endometrial malignancies include advanced age, obesity, early menarche, late menopause, hypertension (HTN), diabetes mellitus (DM), and nulliparity [6]. Endometrial carcinoma is associated with other cancers, such as cervical, vaginal, ovarian, and vulval cancers, and presents with PMB in nearly 95% of cases [9,10]. According to the Indian Cancer Registry, there has been an increasing trend for corpus uteri malignancies since 2000 in postmenopausal women [11].

Cervical carcinoma is the fourth most common cancer in the world. It infrequently presents as PMB; however, high-risk human papillomavirus has been found in cervical cancer screening in postmenopausal women [12]. Various patient characteristics such as multiparity, early marriage, and multiple sexual partners are risk factors for cervical cancer [13]. India accounts for nearly 25% of the worldwide burden of cervical cancer [14]. This study was undertaken to evaluate the various causes of PMB in patients visiting a tertiary care government hospital in northwestern Punjab. Existing literature on women with PMB in this region is scarce, and our results will help to improve the overall standards of diagnosis and treatment of women presenting with PMB. We also aimed to identify applicable clinical criteria that can lead to early detection of malignancy in these patients.

## Materials and methods

This prospective observational study included 120 patients presenting with PMB at our institute during 2020-2021. The patients had their last menstrual period 1 year or more prior to their presentation. A detailed history was obtained from each patient including her name, age, marital status, parity, and socioeconomic status according to the modified B.G. Prasad classification of 2021 (Table 1). This classification is used extensively in India and is a scale based on per capita monthly income.

**Table 1:** The modified B.G. Prasad Classification

Social Class	Per Capita Monthly B.G. Prasad 1961	MODIFIED B.G. Prasad 2021 (INR/month)
Upper Class	>100	7,770 and above
Upper Middle Class	50-99	3,808-7,769
Middle Class	30-49	2,253-3,808
Lower Middle Class	15-29	1,166-2,253
Lower Class	<15	<1,166

The sampling technique chosen was consecutive (non-probability) sampling. Women were excluded from the study if they were on hormone replacement therapy or anticoagulants, were in the perimenopausal age group with abnormal uterine bleeding, or already diagnosed with a gynecological malignancy.

Methodology

Details regarding vaginal bleeding were recorded for each participant, including timing of onset, duration, and amount of bleeding. A detailed clinical history was done, covering co-occurring symptoms such as the presence of vaginal discharge, abdominal pain, abdominal distension, and recent weight loss. Medication history was noted, with particular attention to the use of anticoagulants, hormone replacement therapy, and tamoxifen therapy. All participants underwent a thorough clinical examination. Their height and weight were measured, their body mass index (BMI) was calculated, and their blood pressure was recorded. Abdominal, speculum, and bimanual pelvic examinations were done for assessment of the cervix and to determine the size, position, and mobility of the uterus. Cervical smears were taken, and each participant underwent a transabdominal or transvaginal ultrasound scan to assess the ET and to detect any other pelvic pathology. Examination under anesthesia, cervical biopsy, endometrial biopsy, polypectomy, or dilatation and curettage procedures were done if clinically indicated.

Statistical Methods

Data were collected, tabulated, and analyzed using SPSS (Statistical Package for the Social Science software version 23.0). Continuous variables are presented as mean and standard deviation (SD), while categorical variables are presented as percentages. Chi-square test was applied to determine significance. A P-value of less than 0.05 was considered statistically significant and less than 0.001 as highly significant; a P-value greater than 0.05 was considered insignificant. Pearson’s correlation coefficient was used to check correlations between clinical and histopathologic findings.

Ethical approval

This study was approved by the Institutional Ethical Committee, Guru Gobind Singh Medical College and Hospital, Faridkot with reference number BFUHS/2K21p-TH/554 dated April 21, 2021.

## Results

**Figure 1:**
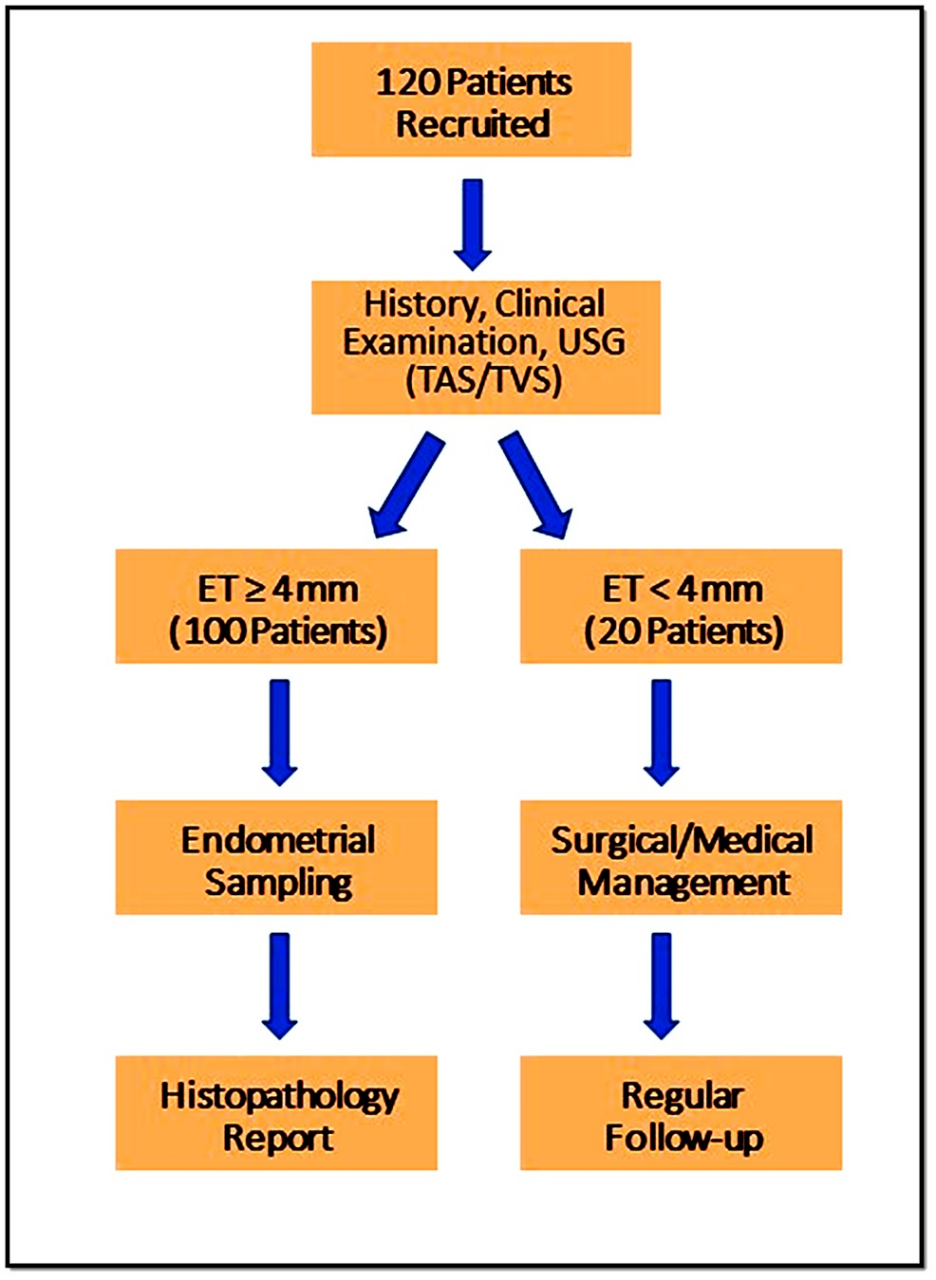
Consort diagram for the study TAS: transabdominal sonography; TVS: transvaginal sonography

As shown in Figure 1, 120 patients with PMB were enrolled in the current study.

Various causes of PMB and the histopathological findings were correlated with various clinical parameters.

Demographics

The age range of patients was 45 to 80 years, with a mean age of 54.97 ± 5.85 years. Fifty-five (54.16%) of the patients were in the age group of 50-54 years and 31 (25.83%) were in the age group of 55-59 years. Sixty-six (55%) of the patients belonged to the middle class of modified B.G. Prasad classification of socioeconomic status and 40 (33.33%) were in the upper middle class. Thirty-seven (30.83%) of the patients presenting with PMB had parity 3, and one patient had parity 8 (0.83%). The average parity was 3.375 ± 1.3 (Tables 1-2).

**Table 2:** Sociodemographic profile

Age Distribution in Range		
Age group (years)	No. of patients (n=120)	Percent (%)
45-49	10	8.33
50-54	55	54.16
55-59	31	25.83
60-64	14	11.66
65-69	7	5.83
70 and above	3	2.5
Socioeconomic Status		
Socioeconomic status (class) – modified B.G. Prasad classification (2021)	No. of patients (n=120)	Percent (%)
Upper Class	11	9.16
Upper Middle Class	40	33.33
Middle Class	66	55.00
Lower Middle Class	3	2.50
Lower Class	0	0.00
Parity		
Parity	No. of patients (n=120)	Percent (%)
1	5	4.16
2	29	24.16
3	37	30.83
4	22	18.33
5	23	19.16
6	3	2.5
8	1	0.83

Clinical characteristics and findings

Thirty-eight (31.66%) of the patients had attained menarche at 13 years followed by 25 (20.83%) at 12 and 14 years each. The mean age of menarche was 13.33 years with a SD of 1.2. Fifty-nine (49.16%) of the patients presented within 1-3 years of menopause, followed by 27 (22.5%) who presented between 4 and 6 years of menopause. On speculum examination, the cervix and vagina were healthy in 65 (54.16%) of the patients with PMB, followed by pale cervix and vagina in 28 (23.33%) and cervical ulcerative lesions in 27 (22.5%). On vaginal examination, the uterus was normal in size in 64 (53.33%) patients (Tables 3-4).

**Table 3:** Menstrual history

Age at Menarche		
Age in years	No. of patients (n=120)	Percent (%)
11	12	10.0
12	25	20.83
13	38	31.66
14	25	20.83
15	20	16.66
Duration of menopause in range (in years)	No. of patients (n=120)	Percent (%)
1-3	59	49.16
4-6	27	22.5
7-9	11	9.16
10 and above	23	19.16

**Table 4:** Examination findings

Per Speculum		
Cervix and Vagina	No. of patients (n=120)	Percent (100%)
Pale	28	23.33
Healthy	65	54.16
Ulcerative lesion	27	22.5
Per Vaginum		
Uterus size	No. of patients (n=120)	Percent (100%)
Normal	64	53.33
Atrophic	12	10.00
Bulky	44	36.66

One hundred (83.33%) patients had an ET ≥ 4 mm and 20 (16.66%) patients had an ET < 4 mm. Six (5%) patients had an ET ≥ 20 mm. Thirty-six (30.00%) of the patients were negative for intraepithelial lesions or malignancy, 10 (8.33%) showed inflammation, and two (1.66%) patients had high-grade squamous intraepithelial lesions and low-grade squamous intraepithelial lesions, respectively. Cervical smear was not done in 62 (51.66%) patients because they had vaginal bleeding at the time of examination (Tables 5-6).

**Table 5:** Endometrial thickness on ultrasonography USG: ultrasonography

USG (Endometrial Thickness in mm)	No. of Patients (n=120)	Percent (%)
1-<4	20	16.66
4-10	54	45.00
11-15	28	23.33
16-20	12	10.00
> 20	6	5.00

**Table 6:** Cervical (Pap Smear) report NILM: negative for intraepithelial lesions or malignancy; LSIL: low-grade squamous intraepithelial lesion; HSIL: high-grade squamous intraepithelial lesion

Report	No. of Patients (n=120)	Percent (%)
NILM	36	30.0
Inflammation	10	8.33
Atrophic	8	6.66
LSIL	2	1.66
HSIL	2	1.66

**Figure 2:**
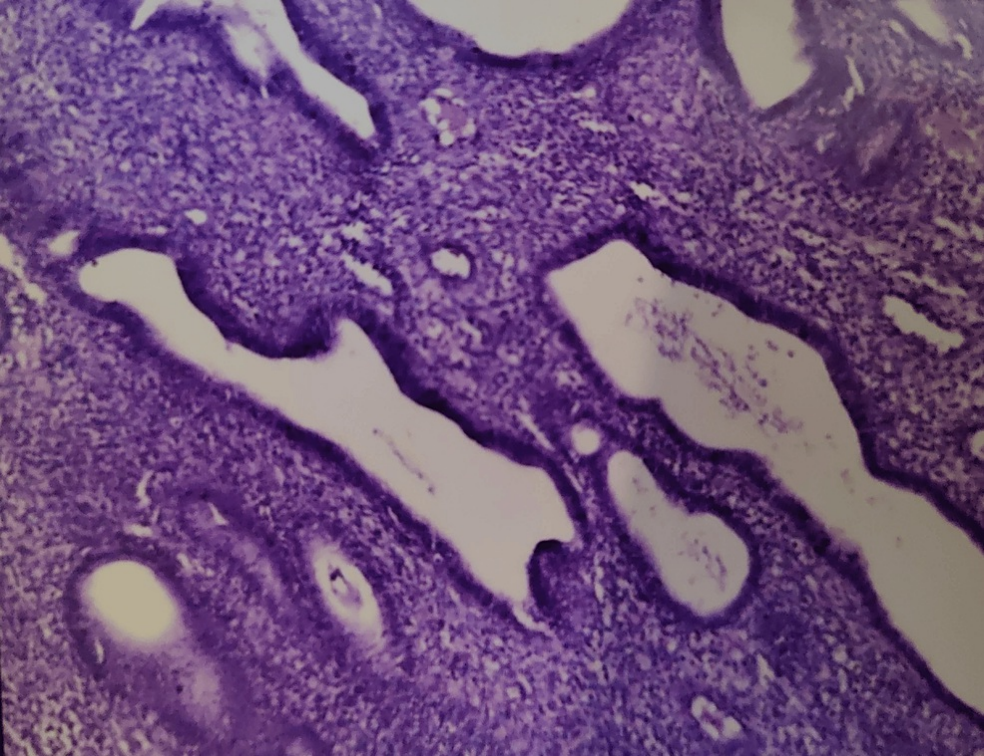
Hematoxylin and eosin-stained histopathology slide showing simple endometrial hyperplasia without atypia.

**Figure 3:**
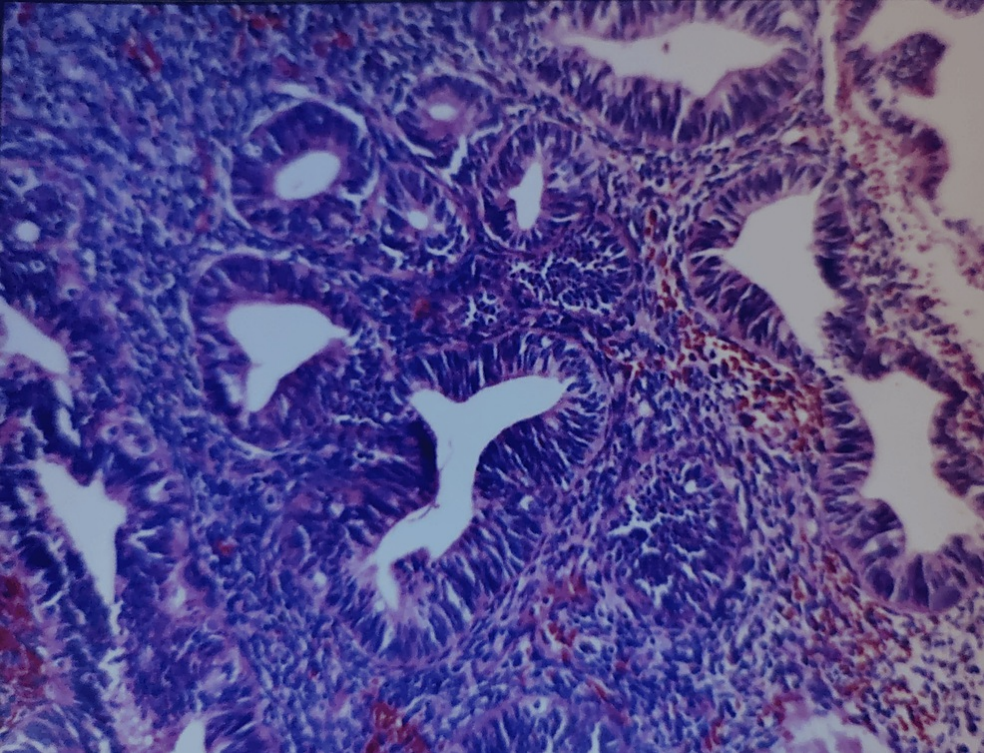
Hematoxylin and eosin-stained histopathology slide showing complex endometrial hyperplasia without atypia.

**Figure 4:**
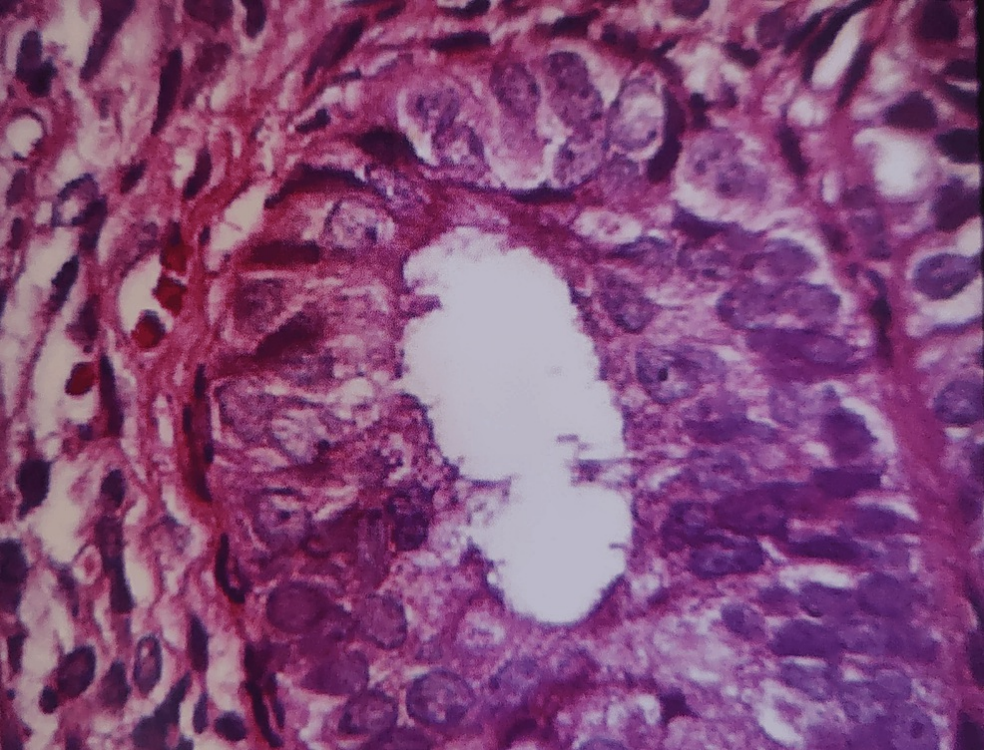
Hematoxylin and eosin-stained histopathology slide showing complex endometrial hyperplasia with atypia.

**Figure 5:**
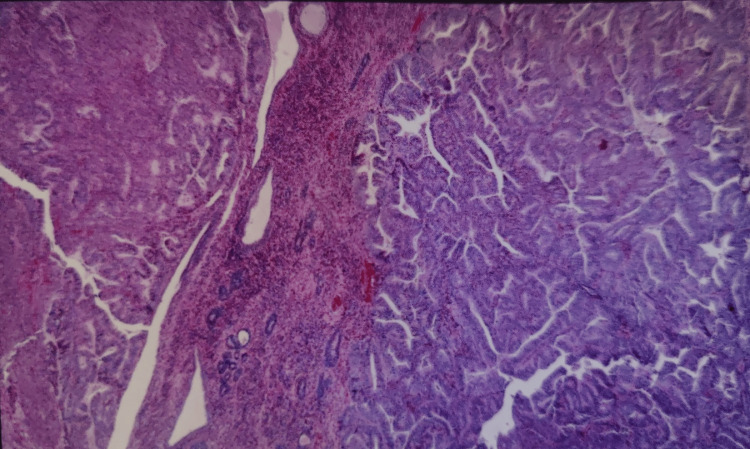
Hematoxylin and eosin-stained histopathology slide showing an endometroid variant of endometrial carcinoma.

Various risk factors in our study were DM in 33 (27.5%), HTN in 28 (23.33%), obesity in two (1.66%), and two or more of these factors in the rest of the patients. An endometrial biopsy was done on 100 patients who had an ET ≥ 4 mm. Endometrial hyperplasia was classified according to the 1994 World Health Organization classification which included Simple hyperplasia without atypia (SHWOA), Complex hyperplasia without atypia, simple hyperplasia with atypia, complex hyperplasia with atypia. SHWOA was seen in 36 (36%) patients followed by 15 (15%) patients with proliferative endometrium. Twelve (12%) patients had endometrial carcinoma (Tables 7-8, Figures 2-5).

**Table 7:** Systemic risk factors HTN: hypertension; DM: diabetes mellitus

Risk Factors	No. of Patients (n=120)	Percent (%)
Nil	42	35
DM	33	27.5
HTN	28	23.33
Obesity	2	1.66
DM+HTN	12	10
HTN + Obesity	1	0.83
HTN + DM + Obesity	2	1.66
Total	120	100.0

**Table 8:** Histopathology findings of endometrial biopsy with endometrial thickness ≥4 mm SHWOA: simple hyperplasia without atypia; SHWA: simple hyperplasia with atypia; CHWOA: complex hyperplasia without atypia; CHWA: complex hyperplasia with atypia

Histopathology Findings	Frequency n=100	Percent (%)
Atrophic	14	14
Proliferative	15	15
Secretory	4	4
Polyp	5	5
SHWOA	36	36
SHWA	6	6
CHWOA	5	5
CHWA	3	3
Endometrial carcinoma	12	12

In 20 patients with an ET < 4 mm, the most common cause of PMB was atrophic endometrium in nine (45%) patients, followed by cervical carcinoma in six (30%) (Table 9).

**Table 9:** Causes of PMB with endometrial thickness <4 mm PMB: postmenopausal bleeding

Causes	No of patients (n=20)	Percent (%)
Atrophic endometrium	9	45
Cervical polyp	2	10
Genital prolapse	2	10
Cervicitis	1	5
Cervical carcinoma	6	30

Comparison of clinical and histopathological characteristics

Fifty-nine (49.16%) patients presented with PMB within 3 years of menopause. Out of these, 28 (47.56%) had SHWOA, while four (6.80%) had endometrial carcinoma. Twenty-seven (22.5%) of the patients with PMB presented 4-6 years after menopause. Eight (29.63%) of these patients had atrophic endometrium, while endometrial carcinoma was found in three (11.11%) of them. Out of 11 (9.16%) patients presenting with PMB 7-9 years after menopause, SHWOA was seen in four (36.6%). In 23 (19.17%) patients presenting ≥ 10 years of menopause, nine (39.13%) had atrophic endometrium and five (21.74%) had endometrial carcinoma (Table 10).

**Table 10:** Duration since menopause and causes of PMB SHWOA: simple hyperplasia without atypia; SHWA: simple hyperplasia with atypia; CHWOA: complex hyperplasia without atypia; CHWA: complex hyperplasia with atypia

Duration of menopause	Causes of PMB													
	Atrophic	Proliferative	Secretory	Uterine Polyp	SHWOA	SHWA	CHWOA	CHWA	Endometrial Cancer	Cancer cervix	Cervicitis	Genital Prolapse	Cervical Polyp	Total
1-3 years	4	12	3	2	28	2	0	0	4	2	0	0	2	59
4-6 years	8	2	1	3	2	3	3	0	3	2	0	0	0	27
7-9 years	2	1	0	0	4	1	1	1	0	0	0	1	0	11
≥10 years	9	0	0	0	2	0	1	2	5	2	1	1	0	23
Total	23	15	4	5	36	6	5	3	12	6	1	2	2	120

Ninety-one (75.83%) of the patients presenting with PMB had benign causes and 29 (24.1%) patients had premalignant or malignant causes. In the benign group, the mean age of patients was 54.15 ± 5.15 years, while in the premalignant/malignant it was 57.82 ± 7.35; the age difference between the two groups was significant (P = 0.005). The average BMI was 21.77 ± 1.80 kg/m^2^ in the benign group, while it was higher (23.62 ± 4.83 kg/m^2^) in the premalignant/malignant group; this difference was also significant (P = 0.001). The age of menarche was 13.24 ± 1.21 years in the benign group and significantly lower (at 12.74 ± 1.18 years) in the premalignant/malignant group (P = 0.08). The parity was 3.20 ± 1.64 in the benign group, while it was significantly higher (at 3.96 ± 1.50) in the premalignant/malignant group (P = 0.007). The ET was 9.47 ± 4.80 mm in the benign group and significantly greater at 11.96 ± 7.41 mm in the premalignant/malignant group (P = 0.018) (Tables 11-12).

**Table 11:** Causes of PMB as benign or premalignant/malignant lesions PMB: postmenopausal bleeding; LSIL: low-grade squamous intraepithelial lesion; SHWOA: simple hyperplasia without atypia; HSIL: high-grade squamous intraepithelial lesion; SHWA: simple hyperplasia with atypia; CHWOA: complex hyperplasia without atypia; CHWA: complex hyperplasia with atypia

Causes	No. of Patients	Percentage (%)
Benign: Atrophic, proliferative, secretory, SHWOA, CHWOA, uterine polyp, cervicitis, cervical polyp, genital prolapse	91	75.8
Premalignant/Malignant: SHWA, CHWA, endometrial carcinoma, LSIL, HSIL, cancer cervix	29	24.1

**Table 12:** Clinical characteristics (quantitative) in benign and premalignant/malignant groups BMI: body mass index

Characteristic	Benign	Premalignant/Malignant	p-value
Mean age (years)	54.15 ± 5.15	57.82 ± 7.35	0.005
Socioeconomic status (Average income in Rupees)	3529.03 ± 1759.09	4179.63 ± 3608.46	0.21
BMI (kg/m^2^)	21.77 ± 1.80	23.62 ± 4.83	0.001
Age of menarche (years)	13.24 ± 1.21	12.74 ± 1.18	0.08
Parity	3.20 ± 1.64	3.96 ± 1.50	0.007
Age of menopause (years)	49.02 ± 1.82	49.44 ± 2.0	0.23
Endometrial thickness (mm)	9.47 ± 4.80	11.96 ± 7.41	0.018

The average age of patients with endometrial carcinoma was 65.5 ± 6.8 years, whereas patients with cervical carcinoma had an average age of 60.5 ± 5.85 years; the difference between the groups was nonsignificant (P = 0.132). The average ET in patients with endometrial carcinoma (17.17 mm) was significantly greater than in patients with cervical carcinoma (2.5 mm) with a significant P-value of 0.00003. In addition, BMI was significantly higher (25.86) in patients with endometrial carcinoma than in patients with cervical carcinoma (21.68) with a significant P-value of 0.05 (Table 13).

**Table 13:** Comparison of clinical characteristics in endometrial and cervical carcinoma causing PMB BMI: body mass index; PMB: postmenopausal bleeding

	Endometrial Carcinoma (mean±SD)	Cervical Carcinoma (mean±SD)	p-value
Age	65.5 ± 6.8	60.5 ±5.85	0.132
Parity	4.4 ± 1.67	3.83 ± 0.98	0.36
Endometrial thickness (ET)	17.17 ± 5.73	2.5 ± 5.85	0.000003
Age of menopause	49.33 ± 1.92	48.67 ± 2.16	0.53
Age of menarche	12.58 ± 1.44	12.67 ± 0.82	0.88
BMI	25.86 ± 6.22	21.68 ± 1.54	0.0465

**Figure 6:**
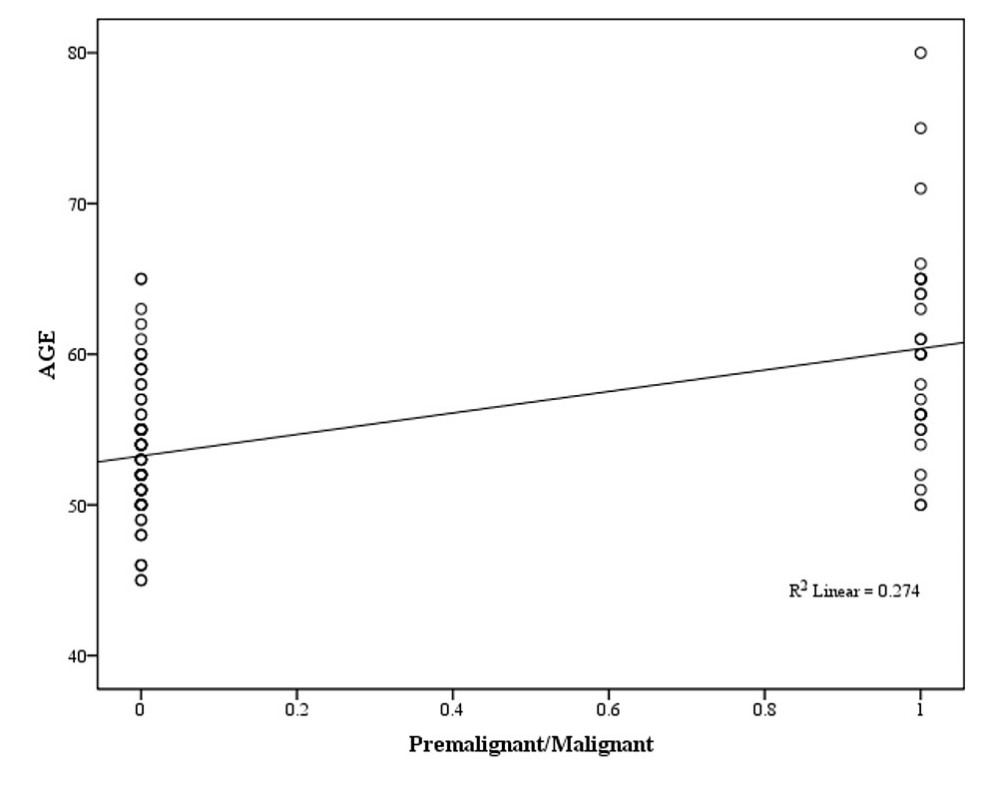
Age correlation with premalignant/malignant pathology (Pearson correlation coefficient)

**Figure 7:**
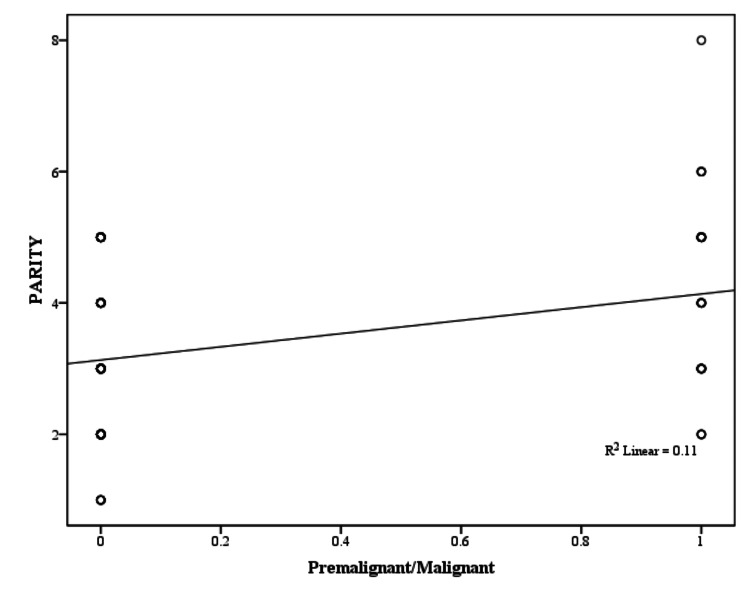
Parity correlation with premalignant/malignant pathology (Pearson correlation coefficient)

**Figure 8:**
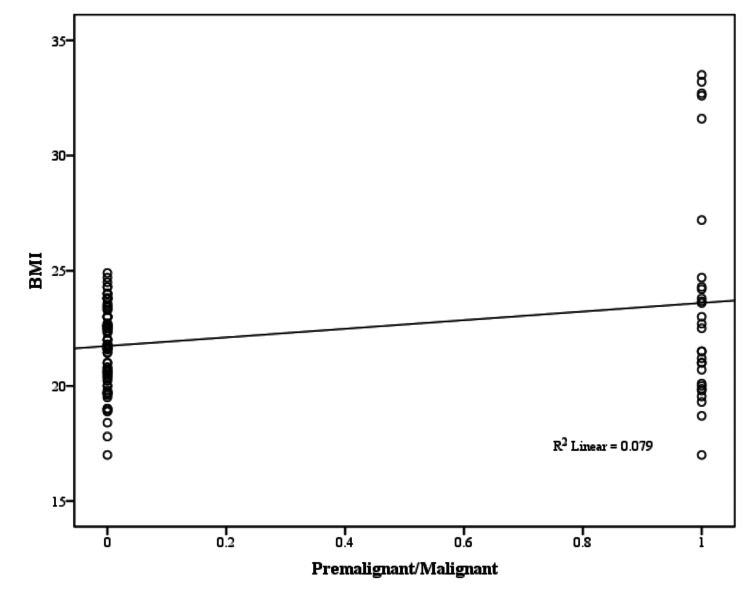
BMI correlation with premalignant/malignant pathology (Pearson correlation coefficient) BMI: body mass index

In the present study, a weakly positive, yet significant (P < 0.05) correlation was observed between the presence of malignancy and increasing age (Pearson correlation coefficient, r = 0.263), parity (r = 0.244), and BMI (r = 0.272), as illustrated in Figures 6-8 and Table 14.

**Table 14:** Clinical characteristics correlation with premalignant/malignant pathology BMI: body mass index

Clinical Characteristics	Pearson Correlation Coefficient (r)	p-value	Significance
Age (years)	0.263	0.004	Positive correlation, chances of malignancy increase with increasing age
Age of menarche (years)	-0.175	0.056	No significant correlation
Parity	0.244	0.007	Positive correlation, chances of malignancy increase with increasing parity
Age of menopause(years)	0.095	0.301	No significant correlation
BMI (kg/m^2^)	0.272	0.003	Positive correlation, chances of malignancy increase with increasing BMI

## Discussion

In our study, the majority of women presenting with PMB were in the age group of 50-54 years, and the mean age of presentation was 54.97 ± 5.85 years with a range of 45-80 years. In a previous study, Begum and Samal included participants ranging from 45 to 80 years with a mean age of 57.17 ± 7.11 years [8]. In a study by Escoffery et al., the mean age of participants was 63.6 years [15]. The age of participants in these studies was similar to that in our study.

The mean age of menopause in participants in our study was 49.1 ± 1.3 years, with almost one-half of participants with PMB (49.16%) being within 1-3 years of menopause. Similar results were reported in a study by Sindhuri and Dongre in which the majority of the cases (53.33%) presented within 1-5 years of menopause [16]. The average parity of participants in our study was 3.375 ± 1.3, with the majority of participants having parity in the range of two to five. Similarly, in a study by Jo et al., the parity was 3.71 ± 1.59 in the group of women < 65 years, while the group with women > 65 years had a parity of 2.27 ± 0.97 [17].

In an evaluation of risk factors, we found that 35% of the patients with PMB in our study had no risk factors, while the rest of the patients had a history of DM (27.5%), HTN (23.33%), DM+HTN (10%), obesity (1.66%), HTN+ DM+ obesity (1.66%), or HTN + obesity (0.83%). These results were similar to the study done by Begum and Samal in which DM was seen in 29% of the participants, HTN in 20%, DM+HTN in 13%, obesity in 3%, and HTN +DM + obesity in 2.5% [8,14]. In a different study, Jo et al. found that 38.24% and 19.05% of participants had HTN, while 22.06% and 8.84% had DM as systemic risk factor in the group of women < 65 years and > 65 years, respectively [17].

Various guidelines recommend endometrial sampling only when the ET is above a certain cut-off level and different guidelines use different ET cut-offs varying from 3 mm to 5 mm. ET cut-off value has been taken as 4 mm in this study based on the study done by Ciatto et al. in which an ET of ≥4 mm had a sensitivity of 91.1% and a specificity of 79.8% for detecting an endometrial carcinoma [18]. The majority of the cases in our study (83.33%) had ET ≥ 4 mm, while only 16.66% had ET < 4 mm. The mean ET was 10.03 mm with an SD of ±5.56 mm. Bruchim et al. found that the mean ET was significantly lower in the absence of endometrial carcinoma (6.9 ± 4.3 mm) than in its presence (13.5 ± 7.7 mm) [19]. Thus, monitoring of ET in high-risk cases can aid in screening for endometrial cancer.

Gredmark et al. reported that the most common cause of PMB was atrophic endometrium (49.9%) in their study [20]. In our study, the most common cause of PMB was SHWOA (30%), followed by atrophic endometrium (19.1%). Proliferative and secretory endometrium was present in 4.2% and 1.3% of cases respectively, in the study by Gredmark et al. [20]. In our study, the incidence of proliferative and secretory endometrium was 12.5% (n = 15) and 3.33% (n = 4), respectively (Table 15).

**Table 15:** Causes of PMB in comparison to other studies SHWO: simple hyperplasia without atypia; SHWA: simple hyperplasia with atypia; CHWOA: complex hyperplasia without atypia; CHWA: complex hyperplasia with atypia; HSIL: high-grade squamous intraepithelial lesion; Ca cervix: carcinoma cervix

Causes	Current study	Gredmark et al. [20]	Singh P et al. [4]	Bafna et al. [21]
Atrophic	34.9%	49.9%	38.33%	48.9%
Proliferative		4.2%		
Secretory		1.3%		
Uterine polyps	4.16%	9.2%	10%	24.4%
Cervical polyps	1.66%			
SHWOA	30%	10%	34.3%	
SHWA	5%			
CHWOA	4.16%			
CHWA	2.5%			
Endometrial carcinoma	10%	8.1%	13.3%	4.08%
Ca cervix	5%	1.31%		
HSIL				
Endometritis				22.4%

The majority of endometrial carcinoma cases (66.66%) in our study were in the age group of 60-69 years, accounting for 10% of the cases of PMB. A similar incidence of endometrial carcinoma was reported by Brand [22]. In the study by Gredmark et al., the majority of cases of endometrial carcinoma occurred in patients who were 65-69 years old, similar to the results of the present study [20]. Ferrazzi et al. reported a peak incidence of 11.5% of endometrial carcinoma in the age group of 56-65 years [23]. The differences between studies could be due to loco-regional and socioeconomic variations in the study populations as they are considered to be independent risk factors for any gynecological malignancy.

All cases of endometrial carcinoma in our study had ET ≥ 4 mm, with a mean ET of 15.36 ± 6.9 mm. Similar results were found in a study by Bruchim et al., who reported that the mean ET was significantly higher (P = 0.005) in the presence of endometrial carcinoma (13.5 ± 7.7 mm) than in its absence (6.9 ± 4.3 mm) [19]. None of the patients in their study had endometrial carcinoma with an ET of <5 mm in comparison with 18.5% of the cases who developed carcinoma when the ET exceeded 9 mm.

The ratio of incidence of endometrial carcinoma to cervical carcinoma among cases with PMB was 2:1 in our study, whereas it was 6.23:1 (8.1% endometrial carcinoma to 1.31% cervical carcinoma) in a study by Gredmark et al. [20]. This difference can be explained by Western countries having a higher incidence of endometrial carcinoma compared with cervical carcinoma, which is the most common gynecological malignancy in women in our region. A Pap smear was not done in 51.66% of patients in our study because they presented with vaginal bleeding at the time of examination. For best results, a Pap smear test should not be performed when a woman is bleeding because the additional cells can obscure cervical cells and alter the test results [24].

The mean age of participants was 54.15 ± 5.15 years in cases of PMB with benign etiology and 57.82 ± 7.35 years in those with malignant etiology and had a significant P-value of 0.005. Thus, it can be inferred that there was a significant difference in age of presentation among patients with malignant etiology presenting at higher age. In the analysis of clinical characteristics and premalignant/malignant etiology, a weakly positive correlation was seen r =0.263; P = 0.004, whereas Jillani et al. did not find any significant association [25]. The positive correlation in our study signifies that as age increases, the chances of malignancy increase.

In our study, the parity of participants with benign causes of PMB was 3.20 ± 1.64 years, while it was significantly higher (P = 0.007) at 3.96 ± 1.50 in the premalignant/malignant group. These findings are consistent with those reported by Jillani et al. who found a significant association between multiparity and malignancy [25]. This finding shows that the chances of malignancy in cases of PMB increase with an increase in parity.

In our study, the average ET was 17.17 mm in patients with endometrial cancer, whereas it was 2.5 mm in cases with cervical cancer. This difference was highly significant (P = 0.000003), and it shows that the risk of having endometrial cancer increases with an increase in ET. Epstein et al. found that the risk of cervical cancer may be even higher in women with PMB who have low ET because the cause of bleeding in these women is less likely to be found in the endometrium [26]. The authors further concluded that cervical cancer was twice as common as endometrial cancer in women having PMB with thin endometrium. These findings are consistent with the results of our study.

A potential limitation of our study is the small sample size. We recommend larger multi-institutional studies to further validate the results of our study.

## Conclusions

In postmenopausal women, any bleeding is considered abnormal. PMB can be due to benign or malignant causes, with benign causes being more common. A common cause of PMB is SHWOA, while endometrial carcinoma is the most common malignant cause of PMB. The incidence of endometrial carcinoma increases with an increase in ET and with an increase in the number of years since menopause. Histopathological examination remains the standard criterion for the correct diagnosis. We recommend initiatives for increasing awareness among the population so that women with PMB can seek prompt medical attention and attain a better prognosis.
